# The Citrus Flavone Nobiletin Reduces Pro-Inflammatory and Pro-Labour Mediators in Fetal Membranes and Myometrium: Implications for Preterm Birth

**DOI:** 10.1371/journal.pone.0108390

**Published:** 2014-09-19

**Authors:** Carrington J. Morwood, Martha Lappas

**Affiliations:** 1 Mercy Perinatal Research Centre, Mercy Hospital for Women, Heidelberg, Victoria, Australia; 2 Obstetrics, Nutrition and Endocrinology Group, Department of Obstetrics and Gynaecology, University of Melbourne, Victoria, Australia; Shanghai Jiaotong University School of Medicine, China

## Abstract

Spontaneous preterm birth is the leading cause of infant death and of neurological disabilities in survivors. A significant proportion of spontaneous preterm births are associated with infection. Infection activates inflammation which induces a cascade of events that leads to myometrial contractions and rupture of fetal membranes. In non-gestational tissues, the citrus flavone nobiletin has been shown to exert potent anti-inflammatory properties. Thus, in this study, we sought to determine the effect of nobiletin on pro-inflammatory mediators in human fetal membranes and myometrium. Human fetal membranes and myometrium were treated with bacterial endotoxin lipopolysaccharide (LPS) in the absence or presence of nobiletin. In addition, the effect of nobiletin in fetal membranes taken from spontaneous preterm deliveries with and without infection (i.e. histological chorioamnionitis) was also examined. In human fetal membranes and myometrium, nobiletin significantly decreased LPS-stimulated expression of pro-inflammatory cytokines (TNF-α, IL-1β, IL-6 and IL-8) and MMP-9 expression and pro-MMP-9 secretion. Additionally, nobiletin significantly decreased COX-2 expression and subsequent prostaglandin (PG) E_2_ production. Notably, nobiletin was also able to reduce the expression and production of pro-inflammatory cytokines and MMP-9 in fetal membranes taken from women after spontaneous preterm birth. In conclusion, our study demonstrates that nobiletin can reduce infection-induced pro-inflammatory mediators in human fetal membranes and myometrium. These in vitro studies further support the increasing volume and quality of evidence that high fruit and vegetable intake in pregnancy is associated with a decreased risk of adverse pregnancy outcomes.

## Introduction

Preterm birth is defined clinically as being born before 37 weeks, or less than 259 days of gestation. There are two main types of preterm birth: spontaneous preterm birth and iatrogenic or medically indicated preterm birth - due to complications in pregnancy such as fetal growth restriction or destabilising preeclampsia [1]. Spontaneous preterm birth accounts for up to 70% of all preterm births, comprising both idiopathic preterm labour and births following preterm pre-labour rupture of membranes (PPROM)). The rate of spontaneous preterm birth has remained static for over a decade, and while tocolytic therapy may successfully delay delivery, these benefits have not translated into improvements in long term neonatal morbidity or mortality [2]. Survivors of preterm birth have greatly increased rates of long term disabilities including cerebral palsy, intellectual handicap and chronic lung disease requiring oxygen [3]. Even moderate degrees of preterm birth have been associated with significant childhood sequelae [4]. Such complications lead to long term morbidity through childhood and extend into adult life, with the attendant financial costs to the health system and incalculable financial and emotional stress to families caring for them [5], [6].

The onset of labour is a complex process involving a myriad of factors, many of which are not yet to be fully defined. No matter if preterm or full-term, there are three common terminal pathways in which the mother prepares for labour, these are: cervical ripening, myometrium contractions, and rupture of the fetal membranes [7], [8]. In the case of spontaneous preterm birth, particularly very early preterm birth, infection is thought to be the biggest aetiological factor [9]–[11]. Bacterial endotoxins bind to cervical and fetal membrane toll like receptors, which in turn promote the production of the pro-inflammatory cytokines TNF-α and IL-1β. These cytokines recruit more TNF-α and IL-1β in a positive feedback loop to sustain the inflammatory response and secondly, they induce other pro-inflammatory and pro-labour mediators that are responsible for triggering the terminal processes of labour [12]–[18]. These mediators include i) the chemokine IL-8; ii) cyclooxygenase-2 (COX-2), responsible for controlling the rate of prostaglandin production that in turn increases myometrium contractility; and iii) extracellular matrix (ECM) remodelling enzymes such as matrix metalloproteinase (MMP)-9, that help breakdown the fetal membranes and remodel the cervix.

Currently there are no effective treatments to prevent or delay spontaneous preterm birth [19]. However, there is now increasing evidence that high fruit and vegetable intake in pregnancy is associated with a decreased risk of adverse pregnancy outcomes [20]–[26]. Many of these beneficial properties have been attributed to phytophenols. In support, we have published that various dietary phytophenols can reduce the mediators involved in preterm labour in human gestational tissues [27]–[30]. Of particular interest are a unique group of phytophenols - polymethoxyflavones - that abundantly exist in the bitter, white pith beneath peels of citrus genus and in smaller amounts in the juices of these fruits. In traditional Chinese medicine, citrus peel has been used to treat and alleviate a wide range of ailments including skin inflammation and respiratory infections for thousands of years [31]. Importantly, and when compared to other phytophenols, citrus flavones have better bioavailability due to higher intestinal permeability and decreased metabolism [32]–[36]. To date, more than 30 citrus flavones have been identified with nobiletin being the most abundant; present in the peels of tangerine, mandarin and oranges. In non-gestational tissues, nobiletin has been shown to possess important biological properties including anti-cancer, anti-inflammatory, anti-diabetic and anti-atherogenic activities [32], [33], [37]–[41]. The aim of this study is to determine the effect of nobiletin on pro-inflammatory mediators in human term fetal membranes and myometrium treated with bacterial endotoxin lipopolysaccharide (LPS), and in fetal membranes after spontaneous preterm birth (with and without chorioamnionitis).

## Materials and Methods

### Ethics Statement

Written informed consent was obtained from all participating patients. Ethics approval was obtained from the Mercy Hospital for Women's Research and Ethics Committee. Pregnant women were recruited to the study by a clinical research midwife.

### Tissue collection

Human placentae with attached fetal membranes and myometrium were obtained from women who delivered singleton infants. Tissues were collected for two studies: from women who delivered at (i) term (>37 weeks gestation) at elective Caesarean section (indications for Caesarean section were breech presentation and/or previous Caesarean section) in the absence of labour; and (ii) preterm (<37 weeks gestation) after spontaneous labour onset. All tissues were obtained within 15 min of delivery.

#### Term studies

Fetal membranes, obtained 2 cm from the periplacental edge, and myometrial biopsies, obtained from the upper margin of the incision made in the lower uterine segment, were obtained from women who delivered healthy, singleton infants from elective Caesarean section in the absence of labour (n = 6 patients). Indications for Caesarean section included repeat Caesarean section or breech presentation. Women with any underlying medical conditions such as diabetes, asthma, polycystic ovarian syndrome, preeclampsia and macrovascular complications were excluded. Additionally, women with multiple pregnancies, obese women, fetuses with chromosomal abnormalities were excluded.

#### Preterm studies

Fetal membranes, obtained 2 cm from the periplacental edge, were obtained from women after spontaneous preterm labour onset (n = 9 patients). The average gestational age for the preterm samples was 29.7±1.3 (range 23.3 to 35.4). All the preterm placentas were swabbed for microbiological culture investigations and assessed for histopathological evidence of infection. Chorioamnionitis was diagnosed pathologically according to standard criteria which included histological evidence of macrophages and neutrophils permeating the chorionic cell layer and often infiltrating the amniotic cell. Four of the cases had histologically confirmed chorioamnionitis from mild to severe; three with PPROM occurring from 5 to 11 days before delivery. The remaining five cases delivered vaginally; three with PPROM occurring from 2 to 26 days before delivery. All the women in the preterm group received antenatal steroids and antibiotics. In addition, five of the women in this study received antenatal magnesium sulfate therapy. None of the women had any underlying medical conditions such as diabetes, asthma, polycystic ovarian syndrome, preeclampsia and macrovascular complications. Additionally, women with multiple pregnancies, obese women, fetuses with chromosomal abnormalities were excluded.

### Tissue explants

**Figure 1:**
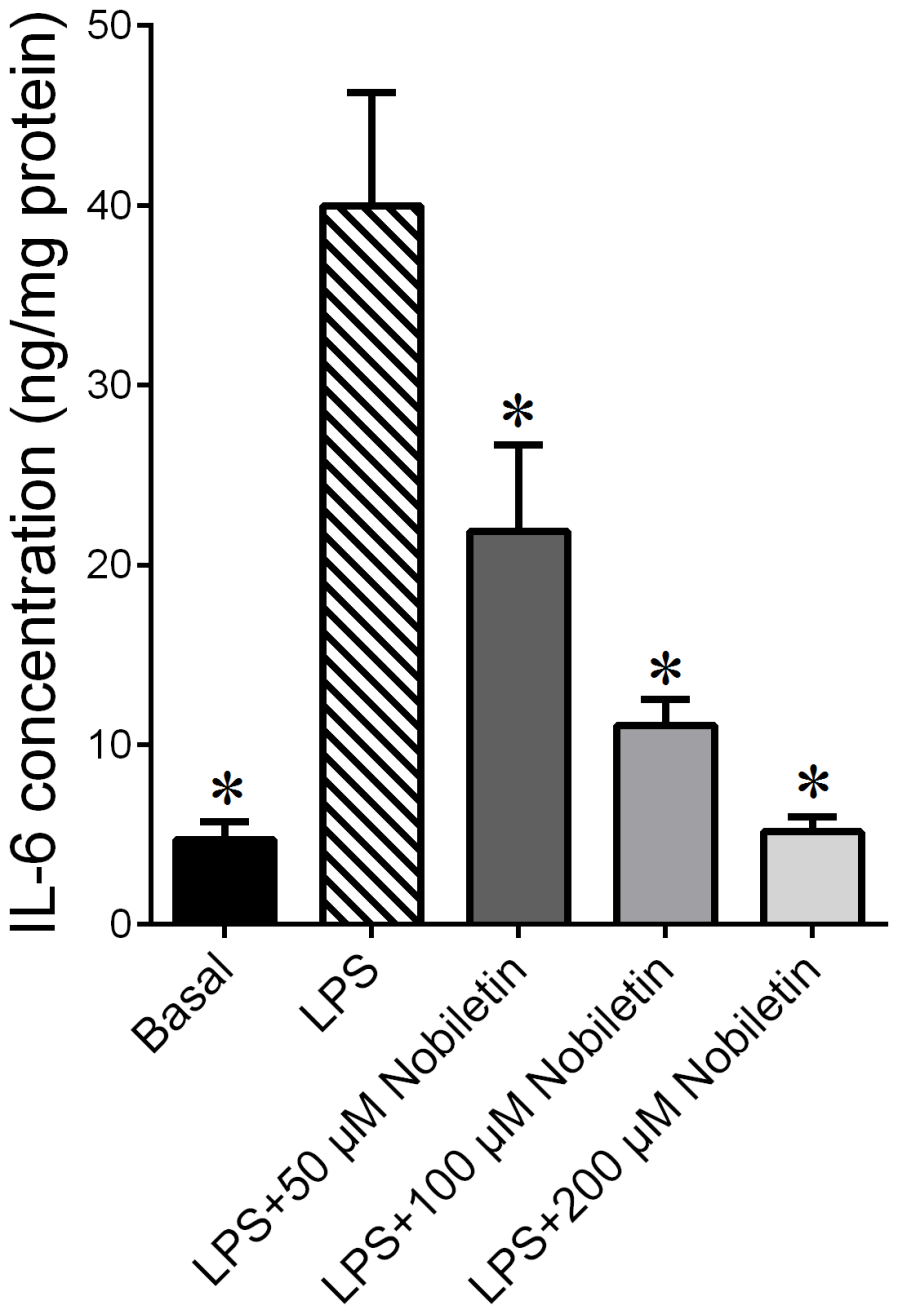
Dose Response: effect of nobiletin on LPS induced IL-6 release from term human fetal membranes. Fetal membranes were incubated with or without 10 µg/mL of LPS in the absence or presence of 50, 100, or 200 µM of nobiletin (n = 6 patients per group). IL-6 concentration in the conditioned media was assayed using ELISA. Each bar shows the mean ± SEM. *P<0.05 vs. LPS (one way ANOVA).

For the term studies, tissue explants were performed as previously described for fetal membranes (combined amnion and choriodecidua) and myometrium [27], [28], [30]. An initial dose response was performed and the data presented in Figure 1. For this study, fetal membranes were incubated in the absence or presence of 10 µg/ml LPS and nobiletin at 50, 100 and 200 µM (Figure 1). While all concentrations of nobiletin decreased LPS-stimulated IL-6 release, treatment with 200 µM nobiletin was closer to basal readings, and was thus used in subsequent experiments. To determine the effect of treatment on cell membrane integrity, the release of the intracellular enzyme lactate dehydrogenase (LDH) into incubation medium was determined as described previously [42]. There was no effect of experimental treatment on LDH activity (data not shown). These data indicate that the concentrations used in this study did not affect cell viability.

For the term explant studies, fetal membranes and myometrium were pre-incubated with 200 µM nobiletin (Life Research; Scoresby, Victoria, Australia) for 1 h, then incubated, for 20 h, in the presence of 10 µg/ml LPS (to facilitate the production of pro-inflammatory mediators). After 20 h incubation, tissue and media were collected separately and stored at −80°C for further analysis as detailed below. Experiments were performed in fetal membranes and myometrium from six patients.

For the preterm study, the effect of nobiletin was determined in fetal membranes after spontaneous preterm labour with and without histological chorioamnionitis (n = 9 patients; n = 5 without histological chorioamnionitis and n = 4 with histological chorioamnionitis). Explants were incubated with or without 200 µM nobiletin for 20 h. After incubation, tissue and media were collected separately and stored at −80°C for further analysis as detailed below. For the preterm studies, due to the large variability in basal release or expression of the endpoints, all data were normalised to the untreated samples (basal), which was set at 1.

### Cytokine and prostaglandin assays

Conditioned medium from tissue culture experiments was assessed for TNF-α, IL-6 and IL-8 concentrations using commercial ELISA according to the manufacturer's instructions (Life Technologies, Mulgrave, Victoria, Australia). The concentration of mature secreted IL-1β in the media was performed by sandwich ELISA according to the manufacturer's instructions (R&D Systems, Minneapolis, MN USA). The concentration of PGE_2_ and PGF_2α_ into the incubation media were assayed using commercially available competitive enzyme immunoassay kits according to the manufacturer's specifications (Kookaburra Kits from Sapphire Bioscience, Waterloo, NSW, Australia). The calculated interassay and intraassay coefficients of variation (CV) were all less than 10%. Data was corrected for total protein and expressed as either ng or pg per mg protein. The protein content of tissue homogenates was determined using BCA protein assay, using BSA as a reference standard, as previously described [43]. For the preterm explant studies, due to patient variability, data were normalised to the untreated samples (basal), which was set at 1.

### Gelatin zymography

Assessment of enzymes of ECM weakening and rupture (MMP-9) was performed by gelatin zymography as previously described [27], [28], [30] on conditioned media collected from the tissue explants. Proteolytic activity was visualised as clear zones of lysis on a blue background of undigested gelatin. For the term explant studies, data were corrected for background, and fold change was calculated relative to LPS, which was set at 1. For the preterm explant studies, due to patient variability, data were normalised to the untreated samples (basal), which was set at 1.

### RNA extraction and qRT-PCR

Analysis of human gene expression by qRT-PCR was performed as we have previously described [27], [28], [30]. Total RNA from cells and tissues was extracted using TRIsure according to manufacturer's instructions (Bioline, Alexandria, NSW, Australia). RNA concentrations were quantified using a spectrophotometer (NanoDrop ND1000, Thermo Fisher Scientific, Waltham, USA). RNA quality and integrity was determined via the A260/A280 ratio. One µg of RNA was converted to cDNA using the SuperScript VILO cDNA synthesis kit (Life Technologies, Mulgrave, Victoria, Australia) according to the manufacturer's instructions. The cDNA was diluted ten-fold and 4 µl of this was used to perform qRT-PCR using SensiFAST SYBR No-ROX kit (Bioline) and 200 nM of pre-designed and validated primers (Qiagen, Chadstone Centre, Victoria, Australia). The specificity of the product was assessed from melting curve analysis. RNA without reverse transcriptase during cDNA synthesis as well as PCR reactions using water instead of template showed no amplification. Average gene Ct values were normalised to the average GAPDH mRNA Ct values of the same cDNA sample. For the term explant studies, fold differences in target gene expression were determined by the comparative Ct method, relative to LPS treatment, which was set at 1. For the preterm explant studies, due to patient variability, data were normalised to the untreated samples (basal), which was set at 1.

### Statistical analysis

**Figure 2:**
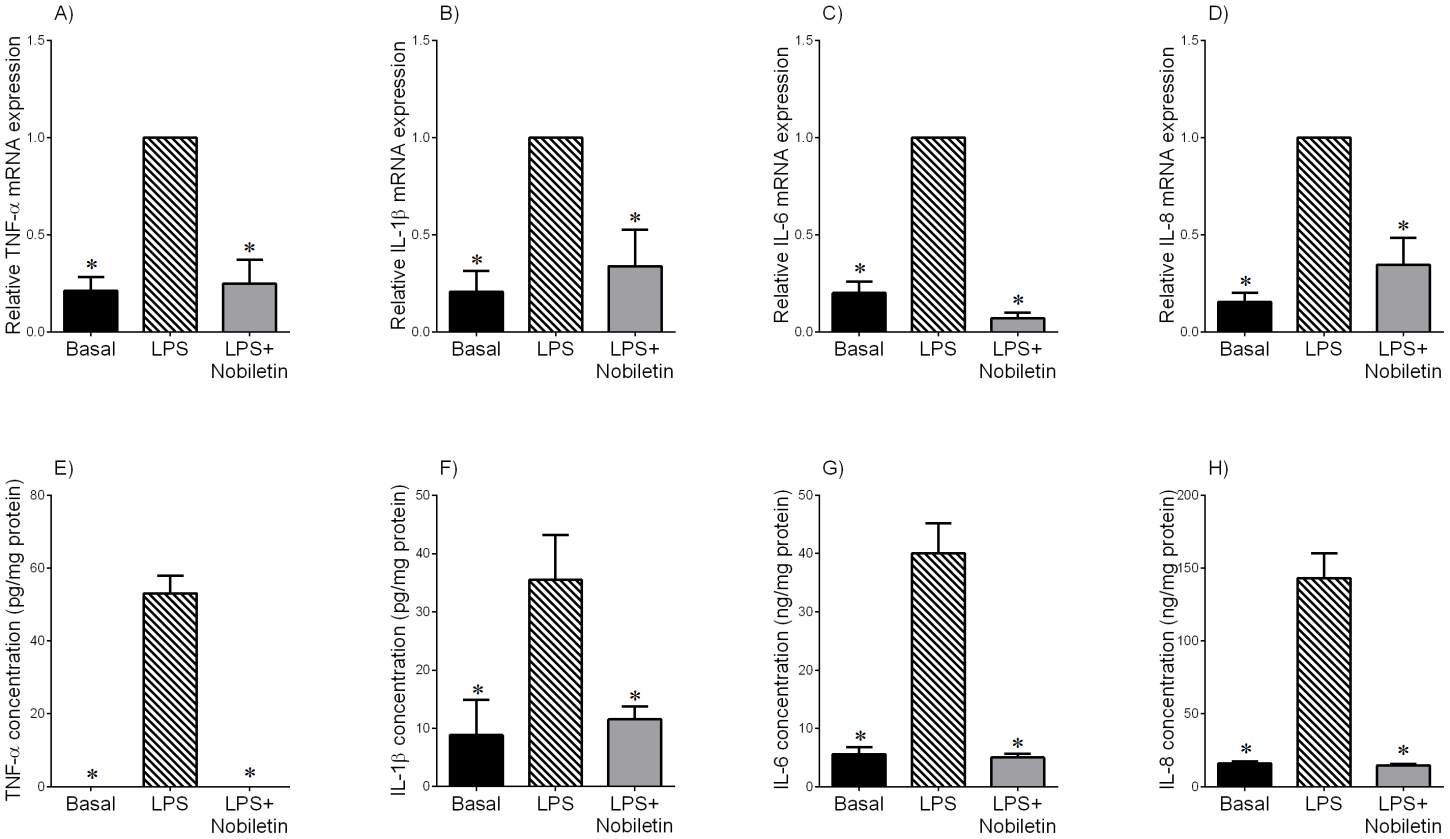
Effect of nobiletin on LPS-induced cytokine expression and release in term fetal membranes. Fetal membranes were incubated with or without 10 µg/mL of LPS in the absence or presence 200 µM of nobiletin for 20 h (n = 6 patients per group). (A–D) TNF-α, IL-1β, IL-6 and IL-8 mRNA expression was analysed by qRT-PCR and normalised to GAPDH mRNA expression. The relative fold change was calculated relative to LPS and data presented as mean ± SEM. *P<0.05 vs. LPS (one-way ANOVA). (E–H) The incubation medium was assayed for concentration of TNF-α, IL-1β, IL-6 and IL-8 by enzyme immunoassay. Each bar represents mean concentration ± SEM. *P<0.05 vs. LPS (one-way ANOVA).

**Figure 3:**
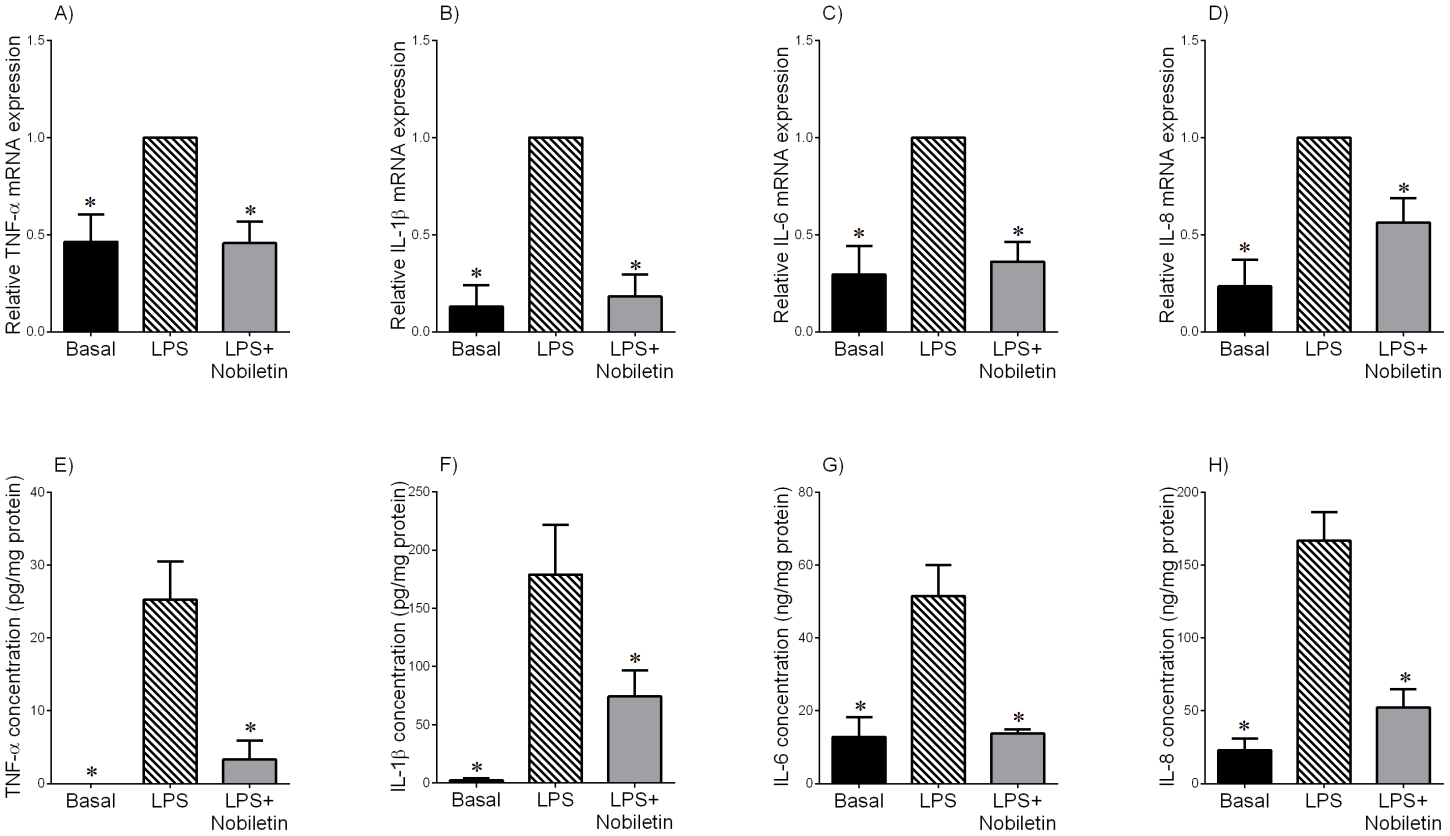
Effect of nobiletin on LPS-induced cytokine expression and release in term myometrium. Human myometrium was incubated with or without 10 µg/mL of LPS in the absence or presence 200 µM of nobiletin for 20 h (n = 6 patients per group). (A–D) TNF-α, IL-1β, IL-6 and IL-8 mRNA expression was analysed by qRT-PCR and normalised to GAPDH mRNA expression. The relative fold change was calculated relative to LPS and data presented as mean ± SEM. *P<0.05 vs. LPS (one-way ANOVA). (E–H) The incubation medium was assayed for concentration of TNF-α, IL-1β, IL-6 and IL-8 by enzyme immunoassay. Each bar represents mean concentration ± SEM. *P<0.05 vs. LPS (one-way ANOVA).

**Figure 4:**
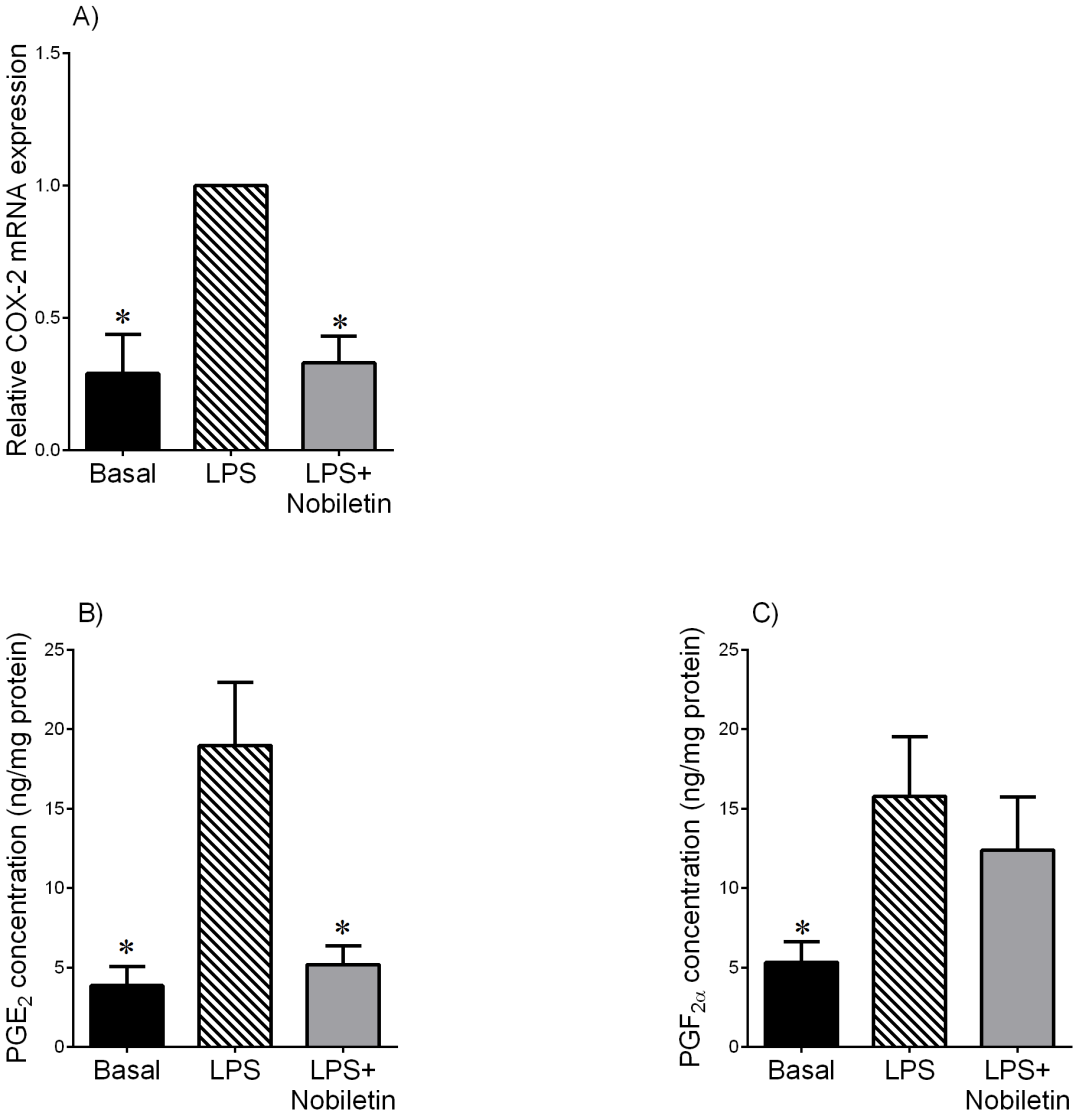
Effect of nobiletin on LPS-induced COX-2 expression and prostaglandin release in term myometrium. Human myometrium was incubated with or without 10 µg/mL of LPS in the absence or presence 200 µM of nobiletin for 20 h (n = 6 patients per group). (A) COX-2 mRNA expression was analysed by qRT-PCR and normalised to GAPDH mRNA expression. The relative fold change was calculated relative to LPS and data presented as mean ± SEM. *P<0.05 vs. LPS (one-way ANOVA). (B,C) The incubation medium was assayed for concentration of PGE_2_ and PGF_2α_ by enzyme immunoassay. Each bar represents mean concentration ± SEM. *P<0.05 vs. LPS (one-way ANOVA).

**Figure 5:**
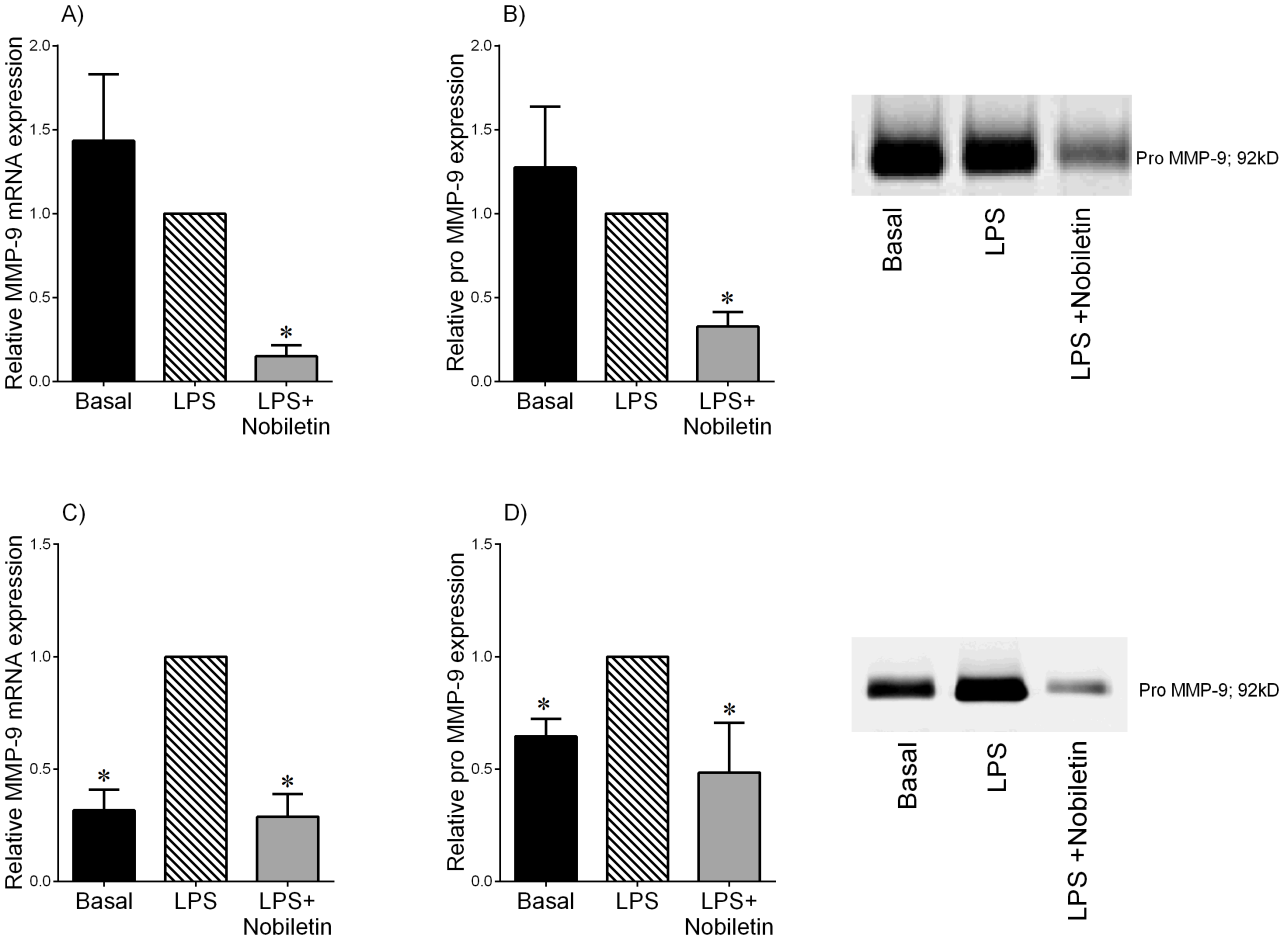
Effect of nobiletin on LPS-induced MMP-9 expression and release in term fetal membranes and myometrium. (A,B) Fetal membranes and (C,D) myometrium were incubated with or without 10 µg/mL of LPS in the absence or presence 200 µM of nobiletin for 20 h (n = 6 patients per group). (A,C) MMP-9 mRNA expression was analysed by qRT-PCR and normalised to GAPDH mRNA expression. The relative fold change was calculated relative to LPS and data presented as mean ± SEM. *P<0.05 vs. LPS (one-way ANOVA). (B,C) The incubation medium was assayed for pro MMP-9 levels by gelatin zymography. The relative fold change was calculated relative to LPS and data presented as mean ± SEM. *P<0.05 vs. LPS (one-way ANOVA). Zymography from one patient per tissue type is also shown.

**Figure 6:**
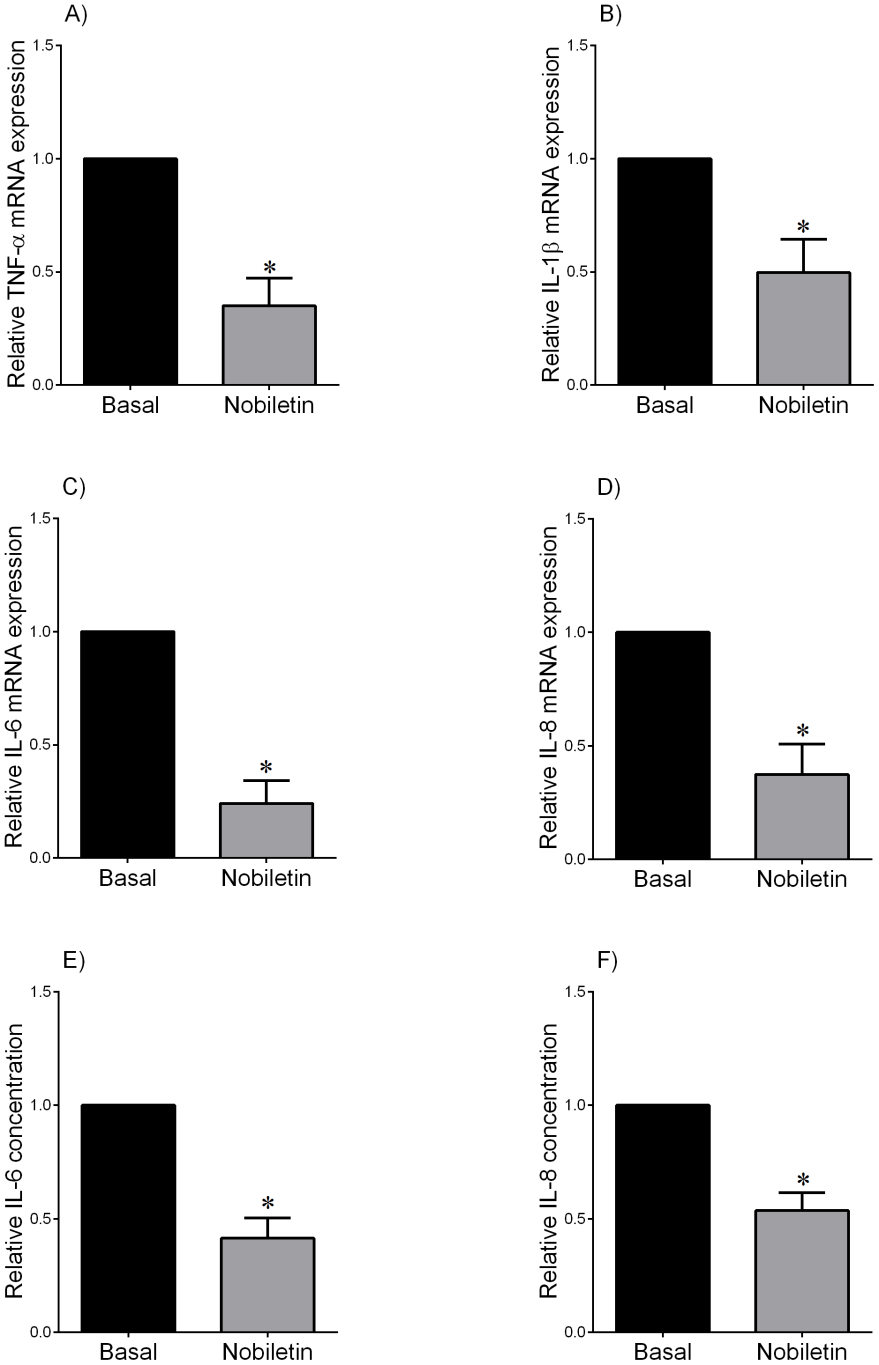
Effect of nobiletin on pro-inflammatory cytokines in preterm fetal membranes. Preterm fetal membranes with histological chorioamnionitis and following spontaneous preterm labour were incubated for 20 h with or without 200 µM nobiletin (n = 9 patients). (A–D) TNF-α, IL-1β, IL-6 and IL-8 mRNA expression was analysed by qRT-PCR and normalised to GAPDH mRNA expression. The fold change was calculated relative to basal expression, which was set at 1. Data is displayed as mean ± SEM (one-way ANOVA). *P<0.05 vs. basal expression. (E, F) The incubation medium was assayed for concentration of IL-6 and IL-8 by ELISA. Data was normalised to untreated (basal) levels, which was set at 1. Each bar represents mean ± SEM (one-way ANOVA). *P<0.05 vs. basal release.

**Figure 7:**
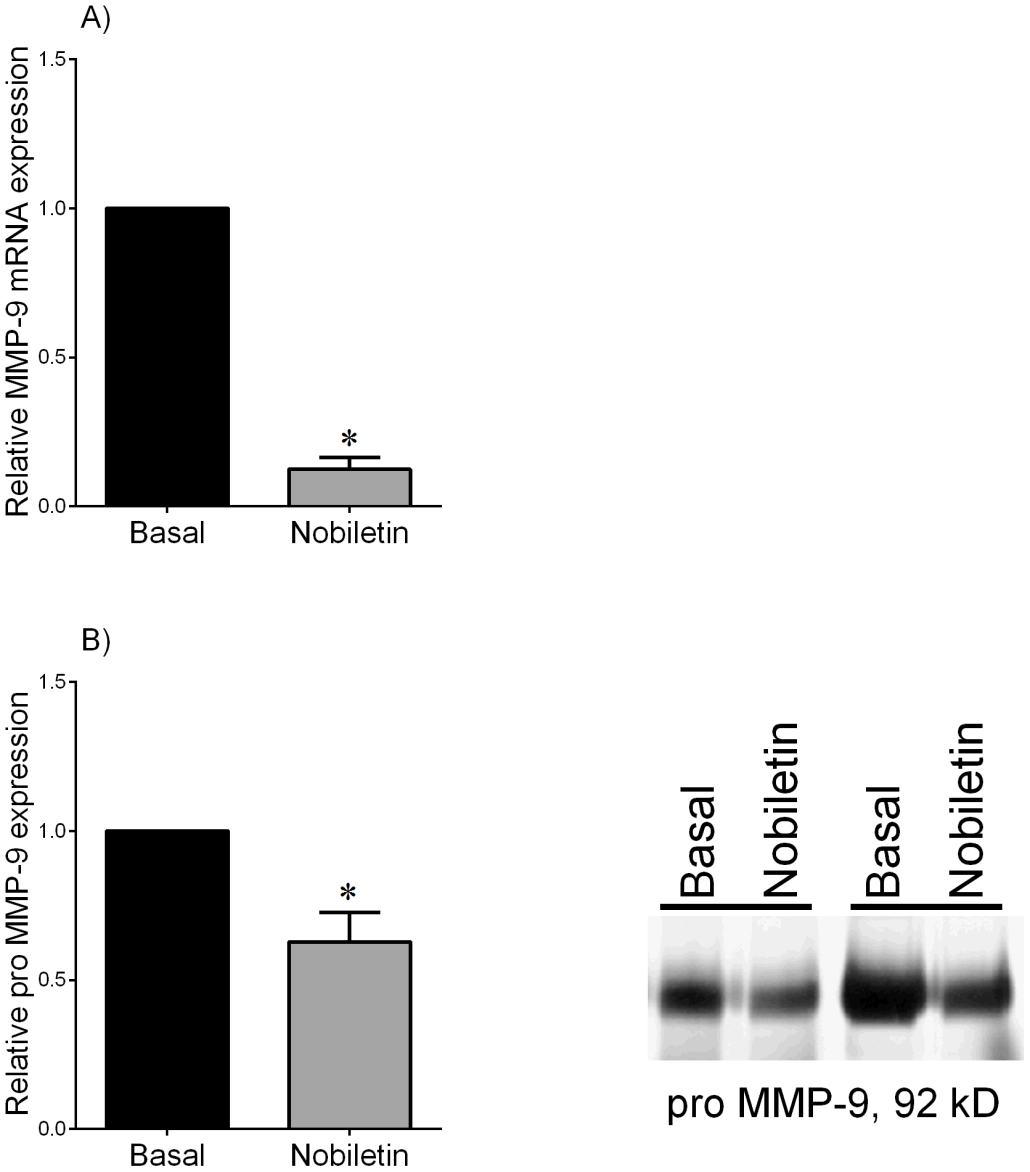
Effect of nobiletin on MMP-9 expression in preterm fetal membranes. Preterm fetal membranes with histological chorioamnionitis and following spontaneous preterm labour were incubated for 20 h with or without 200 µM nobiletin (n = 9 patients). (A) MMP-9 mRNA expression was analysed by qRT-PCR and normalised to GAPDH mRNA expression. The fold change was calculated relative to basal expression, which was set at 1. Data is displayed as mean ± SEM (one-way ANOVA). *P<0.05 vs. basal expression. (B) The incubation medium was assayed for for pro MMP-9 levels by gelatin zymography. Data was normalised to untreated (basal) levels, which was set at 1. Each bar represents mean ± SEM (one-way ANOVA). *P<0.05 vs. basal release. Zymography from two patients is also shown.

Statistics was performed on the normalised data unless otherwise specified. All statistical analyses were undertaken using GraphPad Prism (GraphPad Software, La Jolla, CA). For the term studies (Figures 1, 2, 3, 4, and 5), homogeneity of data was assessed by Bartlett's test; when significant the data was logarithmically transformed before further analysis. The data were analysed by a repeated measures one-way ANOVA and comparisons to the LPS group were performed using the Bonferroni multiple comparison test. For the preterm studies (Figures 6 and 7), a paired Student's t-test was used to assess statistical significance between normally distributed data; otherwise, the Wilcoxon test was used. Statistical difference was indicated by a *P* value of less than 0.05. Data are expressed as mean ± standard error of the mean (SEM).

## Results

### Nobiletin dose response

An initial dose response was performed to investigate whether they would decrease pro-labour mediators, and if so, what dose would be most effective. As shown in Figure 1, LPS induced IL-6 release from fetal membranes. Nobiletin however, significantly decreased LPS induced IL-6 release, with a dose dependent decrease in its concentration (*P*<0.05 for 50 µM, and *P*<0.0001 for 100 µM and 200 µM of nobiletin). Based on these initial studies, 200 µM nobiletin was used for all subsequent experiments in fetal membranes and myometrium.

### Effect of nobiletin on pro-labour mediators in term fetal membranes and myometrium treated with LPS

To examine whether nobiletin would reduce the expression and release of pro-inflammatory and pro-labour mediators in fetal membranes and myometrium, tissues were treated with LPS in the absence or presence of nobiletin for 20 h. Gene expression of TNF-α, IL-1β, IL-6, IL-8, COX-2, and MMP-9 in tissues was assessed using qRT-PCR. Enzyme immunoassays were used to determine the concentrations of pro-inflammatory cytokines (TNF-α, IL-1β, IL-6 and IL-8) and prostaglandin (PGE_2_ and PGF_2α_) in the media. Gelatin zymography was used to examine pro MMP-9 expression.

In fetal membranes, LPS significantly increased TNF-α, IL-1β, IL-6 and IL-8 mRNA expression (Figures 2A–D) and release (Figures 2E–H). Treatment of tissues with nobiletin significantly decreased LPS-stimulated cytokine gene expression and secretion. Similarly, in myometrium nobiletin significantly attenuated LPS-induced TNF-α, IL-1β, IL-6 and IL-8 mRNA expression (Figures 3A–D) and secretion (Figures 3E–H).

The effect of nobiletin on COX-prostaglandin pathway in myometrium is presented in Figures 4A–C; qRT-PCR showed that LPS significantly increased COX-2 mRNA expression from basal (Figure 4A). Nobiletin caused a significant decrease in LPS-induced COX-2 mRNA expression. The release of PGE_2_ and PGF_2α_ into the media was significantly increased by LPS (Figures 4B,C). Nobiletin significantly decreased LPS-induced PGE_2_ release (Figure 4B). However, there was no effect of treatment with nobiletin on PGF_2α_ secretion (Figure 4C).

As we have previously reported, LPS did not significantly increase MMP-9 mRNA expression or pro MMP-9 secretion from fetal membranes (Figures 5A,B). On the other hand, in myometrium, LPS significantly increased MMP-9 mRNA expression (Figure 5C) and pro MMP-9 secretion (Figure 5D). In both tissues, treatment with nobiletin significantly reduced LPS-induced MMP-9 mRNA expression (Figures 5A,C) and secretory pro MMP-9 levels (Figure 5B,D).

### Effect of nobiletin on fetal membranes from spontaneous preterm birth

The above studies demonstrate that nobiletin can significantly reduce pro-inflammatory and pro-labour mediators in term non-labouring fetal membranes and myometrium in the presence of LPS. However, we also wanted to determine if nobiletin could decrease these mediators in tissues from spontaneous preterm birth. For these studies, we used fetal membranes from women with spontaneous preterm deliveries with and without chorioamnionitis. Fetal membranes were treated with or without nobiletin. The effect of nobiletin was found to be equally effective in both non-infected and infected cases, and thus all subsequent data is combined and the data shown in Figures 6 and 7. Treatment with nobiletin significantly decreased TNF-α, IL-1β, IL-6 and IL-8 mRNA expression (Figures 6A–D) and IL-6 and IL-8 secretion (Figures 6E–H) when compared to untreated membranes. Of note, TNF-α and IL-1β secretion could not be measured as the readings were below the sensitivity of the curve. Similarly, nobiletin also significantly decreased MMP-9 mRNA expression (Figure 7A) and secretory levels of pro MMP-9 (Figure 7B).

## Discussion

The majority of preterm births are due to spontaneous preterm birth; that is, spontaneous preterm labour with intact membranes and or preterm pre-labour rupture of membranes (PPROM) [1]. Although there are a number of causes of spontaneous preterm birth, infection and/or inflammation is most commonly associated with preterm birth and thought to have a driving role in PPROM and in initiating uterine contractions [17], [18]. In animal models, LPS is used to model clinical chorioamnionitis given its ability to induce a high-grade intrauterine inflammatory response [44]. Therefore, in this study we utilised LPS to generate a model of chorioamnionitis and spontaneous labour in human myometrium and fetal membranes in order to examine the effect of the citrus flavone nobiletin on pro-inflammatory and pro-labour mediators. In addition, we determined the effect of nobiletin in fetal membranes from spontaneous preterm deliveries with and without histological infection (i.e. chorioamnionitis).

The data presented in this study demonstrate that in human term fetal membranes and myometrium, the citrus flavone nobiletin decreases LPS-induced mRNA expression and secretion of pro-inflammatory cytokines (TNF-α, IL-1β, IL-6 and IL-8), COX-2 mRNA expression and resultant prostaglandin release, and MMP-9 mRNA expression and secretory pro MMP-9 levels. Likewise, in fetal membranes from women with spontaneous preterm labour (with and without infection), nobiletin treatment decreased pro-inflammatory cytokine expression and release, and MMP-9 gene expression and secretory pro MMP-9 levels.

Pro-inflammatory cytokines, produced by macrophages, decidual cells, and fetal membranes in response to bacteria or bacterial products, play a central role in the initiation and progression of human labour and delivery [45]. In this study, we demonstrate that the citrus flavone nobiletin decreases the expression and secretion of pro-inflammatory cytokines TNF-α, IL-1β, IL-6 and IL-8 in term fetal membranes and myometrium stimulated with bacterial endotoxin LPS. Although citrus flavones have not been examined in human gestational tissues before, their anti-inflammatory actions have been demonstrated both *in vitro* and *in vivo* in non-gestational tissues [39], [46]–[50].

Prostaglandins have long been recognised as a key mediator of labour, and are often clinically used to promote cervical ripening [51]. Increased concentrations of prostaglandins and the enzyme COX-2 also occur during infection-induced preterm birth [7], [52]–[56]. Prostaglandins cause preterm birth in the same manner as full term parturition, by inducing the terminal processes of labour: fetal membrane rupture, cervical dilation, and myometrial contractility [57]. They cause membrane rupture by stimulating the ECM remodelling enzyme MMP-9, that in turn leads to cell apoptosis and breakdown of collagen in the fetal membranes [58], [59]. Cervical dilation is achieved by PGE_2_ stimulating collagenolytic activity [51]. Prostaglandins increase uterine contractility by altering the muscles' electro-physiology, making its response to contractile stimulus larger and more coordinated [60]. All prostaglandins are synthesised from arachidonic acid with COX-2 being the rate-limiting enzyme, making it a key indicator of prostaglandin production [61]. In this study, nobiletin decreased LPS-induced COX-2 mRNA expression and PGE_2_ release in myometrium. There was, however, no effect of nobiletin on PGF_2α_ release suggesting that nobiletin does not regulate PGF synthase which converts PGH_2_ to PGF_2α_.

MMPs play a crucial role in preparing the myometrium and fetal membranes for parturition. MMP-9 in particular is up regulated in both myometrium and fetal membranes in both term and preterm birth [62]–[64]. In infection-induced preterm birth, the increase in pro-inflammatory cytokines, chemokines, and prostaglandins all lead to increased expression of MMP-9 [58], [59], [65]. In fetal membranes, MMP-9 degrades the collagen that makes up the extracellular structure [66]–[68]. This degradation weakens the membranes and lead to PPROM [69]. PPROM occurs in between 30–40% of spontaneous preterm birth, and often is associated with a clinical or sub-clinical intra-uterine infection [70]. Normally, labour will follow PPROM however if it does not there is a significant increased risk of acute intrauterine infection [66]. In this study, LPS only increased MMP-9 mRNA expression in the myometrium; however nobiletin decreased MMP-9 mRNA expression and release in both fetal membranes and myometrium.

It is now well-established that spontaneous preterm birth is associated with increased expression and secretion of pro-inflammatory mediators [45]. Therefore, in this study, we also examined if nobiletin could suppress inflammation in fetal membranes taken from spontaneous preterm deliveries with and without histological chorioamnionitis. Notably, we found that nobiletin significantly decreased the expression and release of pro-inflammatory cytokines, and MMP-9 gene expression and secretion of pro MMP-9 in fetal membranes obtained at preterm after spontaneous labour and delivery; both in the absence and presence of chorioamnionitis. These results indicate the potential of the citrus flavones nobiletin as either a part of a dietary intake before PPROM and preterm labour occurs or as a therapy for threatened cases of preterm birth. Indeed, pregnant women consuming a Mediterranean-type diet (>5 fruits or vegetables a day) had a reduced risk of preterm birth when compared to women who did not follow the diet [71]. Additionally, intake of dried fruits, particularly raisins, is associated with a reduced risk of PPROM [72]. Interestingly, raisins contain the phytochemical compounds resveratrol and kaempferol which we have previously shown to possess potent anti-inflammatory activities in human gestational tissues [28], [29].

A number of studies have investigated the effectiveness of several other phytophenols in reducing pro-inflammatory and pro-labour mediators in gestational tissues during infection or inflammation. For example, we have previously reported that curcumin (found in turmeric), naringenin (found in grapefruit and tomatoes), apigenin (found in celery and parsley), luteolin (found in many foods including celery and parsley), kaempherol (found in many food stuffs, including grapefruit and strawberries), resveratrol (found in the skin of red grapes) and silibinin (from milk thistle) exert potent anti-labour activities in human fetal membranes and myometrium [27]–[30]. Collectively, our current and published data provide support for the increasing volume and quality of evidence that high fruit and vegetable intake in pregnancy is associated with a decreased risk of adverse pregnancy outcomes [20], [22], [26], [71], [73].

A major limiting factor in the potency and potential of phytophenols as therapeutic agents is their poor oral bioavailability. However, polymethoxyflavones such as nobiletin, owing to their methylation, have improved transport through biological membranes (such as the intestine) and an increase in oral bioavailability [32]–[34]. In addition, they are active at much lower doses. Thus, attainment of effective *in vivo* concentrations by dietary supplementation may be more plausible. Indeed, supplements containing citrus polymethoxyflavones have shown promising cardioprotective effects in humans [74]. Human studies focussed on dose, bioavailability, efficacy and safety are, however, required to propel the use of these promising therapeutic agents into the clinical arena. Of promise are the studies using Sytrinol for heart disease. Sytrinol is a patented dietary supplement containing the citrus flavones nobiletin and tangeretin at 1∶1 ratio. These studies highlight the translation potential of our findings.

Preterm birth is a global issue that affects the lives of millions of families every year and causes over a million deaths every year [75]. Currently there are no long-term treatments, with most only capable in delaying birth by hours [19]. Inflammation has a central role in the genesis of preterm birth and the adverse neonatal sequelae that follow [3], [76]–[78]. Thus, a safe and effective agent that could block the inflammatory response would be an ideal therapeutic. In this study, we report the beneficial actions of the citrus flavone nobiletin in reducing pro-inflammatory and pro-labour mediators in the presence of bacterial endotoxin LPS. Significantly, we were also able to show that nobiletin can reduce the inflammation already present in the preterm fetal membranes. These studies support the numerous epidemiological studies that high fruit and vegetable intake in pregnancy is associated with a decreased risk of adverse pregnancy outcomes [20]–[26].
